# Challenges in diagnosing canine spindle cell tumours using immunohistochemistry, illustrated by three nonpigmented malignant cases from the nictitating membrane

**DOI:** 10.1186/s13028-024-00727-z

**Published:** 2024-02-23

**Authors:** Kristine Bundgaard Kjellingbro, Carolina Naranjo Freixa, Lauge Hjorth Mikkelsen, Steffen Heegaard

**Affiliations:** 1 Anicura Veterinärhuset Ängelholm, Kristianstadsgatan 1, 26271 Ängelholm, Sweden; 2 IDEXX Laboratories, Carrer Plom 2-8, 3ª, 08038 Barcelona, Spain; 3 Eye Pathology Section, Department of Pathology, Rigshospitalet, University of Copenhagen, Frederik V’s Vej 11, 1st Floor, 2100 Copenhagen Ø, Denmark; 4 Department of Ophthalmology, Rigshospitalet, University of Copenhagen, Frederik V’s Vej 11, 1st Floor, 2100 Copenhagen Ø, Denmark

**Keywords:** Amelanotic melanoma, Dog, Dog-adapted immunohistochemistry protocol, Hemangiosarcoma, Third eyelid

## Abstract

**Background:**

Nonpigmented malignant spindle cell tumours of the membrana nictitans are rare in dogs. In twenty-three years only three cases have been diagnosed in Scandinavia. This study describes the three cases of malignant tumours of the membrana nictitans recorded by the Eye Pathology Section, University of Copenhagen, Denmark, with reference to the clinical appearance and work-up, the treatment and prognosis, and the histopathological description including immunohistochemistry. The three cases are compared to previous publications on canine tumours of the nictitating membrane. We emphasize the importance of using protocols that are adapted to the specific species such as dogs. Opposite the human tissue responses, we even need more than one marker when diagnosing melanomas in dogs.

**Results:**

The dogs presented were an 8-year-old Dachshund, a 12-year-old Akita and a 14-year-old Shetland Sheepdog. All three dogs were entire females. All three nictitating membrane tumours developed on the right nictitating membrane as firm or multilobulated hyperaemic masses. Two of the tumours were macroscopically nonpigmented, the third being partly pigmented on the surface and ulcerated. According to the histopathology and for two of the cases immunohistochemistry with dog-adapted protocols the diagnoses included one hemangiosarcoma and two amelanotic melanomas. Tumour regrowth developed in all three cases and repeated resections were completed 1, 2 and 3 times, respectively, with recurrence experienced within 1.5 months − 3 years.

**Conclusions:**

Nonpigmented malignant spindle cell tumours of the canine membrana nictitans are rare. Treatment of choice should be complete excision with a minimal histologic tumour-free distance and in case of a recurrence a full resection of the nictitating membrane. We strongly recommend a dog-adapted protocol for immunohistochemistry.

## Background

Tumours of the membrana nictitans are quite rare in dogs [1, 2], the most common being adenomas or adenocarcinomas arising in the membrana nictitans gland [2–5]. Other tumours recorded are squamous cell carcinomas [5, 6] and papillomas [3, 7]; melanomas and melanocytomas [3, 8]; hemangiosarcomas [9, 10], hemangiomas [3, 10, 11] and angiokeratomas [12, 13]; leiomyoma [14]; mast cell tumours [15]; lymphomas [16, 17]; plasmacytomas [18] [G. C. Shaw, personal communication, COPLOW, 2019], myoepitheliomas [19, 20], basal cell carcinomas [20–22] and complex carcinomas [20]; a transmissible venereal tumour [23]; a malignant peripheral nerve sheath tumour [24]; and a histiocytoma [3].

Melanomas in general are malignant tumours relatively common in dogs, especially the pigmented types located in the skin [25]. Melanomas of the canine conjunctiva are rare in the literature, most of them being pigmented [3, 8][C. R. Reilly et al., ACVO 2005 abstract no.: 39], making the amelanotic melanomas in the canine nictitating membrane very rare. Melanomas arise from melanocytes which originate from the neural crest [26], they vary histopathologically from epithelioid cell types to spindle cell types or a mix of both [3]. Earlier publications in humans have shown that UV exposure is a risk factor [27], but recent research has suggested multiple causes [28]. Risk factors for canine melanomas are still under research, they develop in the same locations as in humans, but there is a strong breed predisposition and overrepresentation in black coated dogs, associated with both UV and non-UV induced pathways [29].

Sarcomas in general are malignant tumours derived from connective tissues. Hemangiosarcomas are composed of neoplastic endothelial cells [30]. In dogs most sarcomas seem to be spontaneous. Earlier publications strongly indicate that UV exposure is a risk factor in conjunctival hemangiosarcomas [10, 31–33]. There is no breed predisposition for conjunctival hemangiosarcomas, but middle-aged to older, middle to large-size dogs with significant outdoor activity are more commonly affected [9, 10, 30]. Neither is there a sex predisposition [10], but occurrence of hemangiosarcomas in general is more common in neutered individuals as opposed to those that are intact, indicating a possible hormonal link [34].

Tumours of the nictitating membrane are in general characterized by protrusion of a firm or irregular local mass expanding the nictitating membrane, either on the bulbar side, the palpebral side or on the leading edge of the nictitating membrane [4]. Melanomas in the nictitating membrane are mostly heavily pigmented, though they can be even partly or totally amelanotic [35]. Vascular tumours in this area arise in different shades of pink, most often in nonpigmented areas with conjunctival hyperaemia being present [9–11]. For vascular tumours a blood blister-like appearance is also quite typical [34].

Early, complete surgical excision is recommended and may be curative, though recurrence is a risk, as malignant spindle cell tumours in general demonstrate aggressive, local [10, 36] or multi-focal [35] invasive tissue involvement. In human oncology the minimum surgical margin to reduce the risk of local recurrence of sarcomas has not yet been clearly defined [37, 38]. In cutaneous melanomas there are more well-established standards recommending 1 to 2 cm margins depending on the thickness of the primary tumour [39, 40]. In the future, optical coherence tomography (OCT) [41] or micrographic surgery [42] may prove a helpful intra-operative tool for visualizing tumour-affected or tumour-free margins in surgery of dogs.

The treatment of choice for melanomas and hemangiosarcoma of the nictitating membrane in dogs is surgery. Radiation therapy and systemic chemotherapy has been used with success in melanomas and non-dermal hemangiosarcomas [34, 43]. Immunotherapy is being applied for melanomas [44, 45].

Histopathological variation represents a diagnostic challenge in specifying tumour type as many neoplastic cells have histologic patterns with overlapping features, but the development of immunohistochemistry has improved the diagnostic process of spindle cell tumours and especially melanomas in veterinary pathology [46]. Using a dog-adapted protocol is essential to secure the right diagnosis. Most immunohistochemical protocols are developed for human tissues and must be controlled and adapted to each specific species to avoid false positive or negative results.

The aim of this study is to describe three rare cases of malignant macroscopically nonpigmented spindle cell tumours of the canine membrana nictitans and compare to previous publications on this subject. We emphasise the importance of using an immunohistochemistry protocol adapted to dogs.

## Methods

This retrospective study included three canine patients managed clinically by ECVO (European College of Veterinary Ophthalmology) recognised authorized veterinary ophthalmologists. The three malignant spindle cell tumours of the nictitating membrane were diagnosed by co-author SH at the Eye Pathology Section, Copenhagen University Hospital (Rigshospitalet) in Denmark from 2000 to 2023. In twenty-three years only three malignant nonpigmented spindle cell cases have been diagnosed in Scandinavia [S. Heegaard, personal communication, 2023], [R. Grandón, personal communication, BioVet, 2023]. The tumours were further analysed by co-author CNF during 2022 and 2023. This study did not require official or institutional ethical approval. The animals were handled clinically according to high ethical standards and national legislation.

### Histopathology and immunohistochemistry

Archived tissue samples were retrieved in the three cases. All specimens were formalin-fixed and paraffin-embedded (FFPE) and stained with haematoxylin and eosin (H&E), Gram, Trichrome Gomori, Masson trichrome, and periodic acid-Schiff (PAS) according to standard protocols. The FFPE blocks were retrieved and additional, serial 4-µm tissue sections were cut and mounted on slides prior to immunohistochemical staining. Immunohistochemical stains were performed on a Ventana BenchMark ULTRA platform (Ventana Medical Systems Inc., Tucson, AZ, USA) as previously described [47], according to a human protocol. The following primary antibodies were used: S-100 (Polyclonal, 1:4000 dilution, DAKO A/S, Glostrup, Denmark), Vimentin (clone 3B4, 1:400 dilution, DAKO A/S), Cytokeratin (clone AE1/AE3, 1:200 dilution, DAKO A/S), Smooth muscle actin (SMA) (clone 1A4, 1:500 dilution, DAKO A/S), Glial fibrillary acidic protein (GFAP) (polyclonal, ready-to-use (RTU), DAKO A/S) and Melan-A (clone: A103, 1:100 dilution, DAKO A/S). The Dako Envision Flex system Labelled Polymer Anti-mouse (Dako Agilent, Santa Clara, CA, USA) was used as a secondary antibody according to the manufacturer’s instructions. (Table 1).

**Table 1:** Immunohistochemistry protocols

Protocol	Human	Dog-adapted	Dog-adapted
*Target*	Melan-A	Melan-A	PNL2
*Antigen retrieval*	-	EDTA buffer, pH9	Sodium citrate
*Primary antibody*	Melan-A clone 103, Dako (dilution 1:100)	Melan-A clone 103, Dako (dilution 1:50)	PNL2 clone sc-593,006, Santa Cruz (dilution 1:100)
*Secondary antibody*	Envision Flex system labelled polymer anti-mouse	Envision + system-HRP labelled polymer anti-mouse	Envision + system-HRP labelled polymer anti-mouse
*Detection/ chromogen*	DAB chromogen (Dako)	DAB chromogen (Dako)	DAB chromogen (Dako)

Description of the human and the dog-adapted immunohistochemistry protocols used on the current canine membrana nictitans tumours

Additionally, Melan-A and PNL2 antibodies were applied to detect melanocytic differentiation using a protocol that has been standardized for use in canine tissues. Briefly, for Melan-A the Dako clone A103 (Dako Agilent, Santa Clara, CA, USA) was used as a primary antibody at a 1:50 dilution. Antigen retrieval was done with pH9 EDTA buffer (Fischer Scientific, Loughborough, Leicestershire, UK). For PNL2, the Santa Cruz Clone PNL2 sc-59,306 (Santa Cruz Biotechnology, Heidelberg, Germany) was used at a 1:100 dilution. Antigen retrieval was done with sodium citrate buffer (Fischer Scientific, Loughborough, Leicestershire, UK). The Dako Envision + system-HRP Labelled Polymer Anti-mouse (Dako Agilent, Santa Clara, CA, USA) was used as a secondary reagent for both primary antibodies. Primary and secondary antibodies were both incubated for 30 min at room temperature and the reactions were visualized using DAB + substrate buffer (and DAB chromogen Dako Agilent, Santa Clara, CA, USA) for 10 min. (Table 1).

## Results

### Case 1

**Fig. 1:**
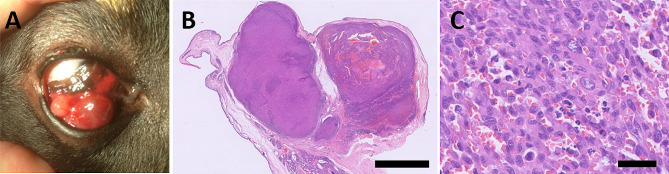
Hemangiosarcoma in the nictitating membrane of an 8-year-old Dachshund. (**a**) Hemangiosarcoma in situ before third resection. The tumour arises from the palpebral side of the nictitating membrane of the right eye and has a multilobulated and cystic appearance. (**b**) Subgross view of the excised nictitating membrane showing a nodular growth in the conjunctival substantia propria on the palpebral side. Hematoxylin and eosin (bar = 2 mm). (**c**) High magnification view of the neoplastic proliferation, showing plump spindle cells lining irregular vascular-like channels. Hematoxylin and eosin (bar = 100 μm)

A 12 kg, 8-year-old intact female Dachshund dog presented with a hematoma-like lesion on the palpebral surface of the right nictitating membrane, the rest of the ophthalmic examination was normal. Initially the hematoma was concluded to be due to trauma but after one week of progressive enlargement, a tumour was suspected. Surgical excision of the mass was performed under general anaesthesia. This mass was not sent for histopathology. The surgical site healed uneventfully but after 1.5 months regrowth was noticed. Another excision was performed under general anaesthesia. Two months later, a further regrowth appeared on the same site. This time the tumour had a multilobulated and cystic appearance (Fig. 1a). A larger resection of the tumour and the nictitating membrane was performed under general anaesthesia. This resection was sent for histopathology. The dog survived for another 3 years and was then euthanized for reasons unrelated to this disease. At this point another regrowth was observed in the remnants of the nictitating membrane. Necropsy was not performed. (Table 2).

**Table 2:** Clinical and histopathological data

Case	Breed	Age	Gender	Lateralization	Tumour presentation and location	Histopathological diagnosis	Surgical treatment	First / Second/ Third recurrence/ prognosis
1	Dachshund	8y	F	OD	Hematoma-like tumour on the palpebral side of NM	Hemangiosarcoma	Surgical resection of the mass, resection of NM at 3rd surgery	1,5 months after 1st surgery/ 2 months after 2nd surgery/ euthanized 3 years later when another recurrence
2	Akita	12y	F	OD	Hyperaemic, grossly nonpigmented tumour on the palpebral side of NM	Sarcoma according to the human protocol, very lightly pigmented melanoma according to the dog-adapted protocol	Surgical resection of the mass, exenteration at 3rd surgery	1,5 years after 1st surgery/ 7 months after 2nd surgery/ 4 months after 3rd surgery/ euthanized 1 months later when another recurrence
3	Shetland Sheepdog	14y	F	OD	Pendulous, on the surface partly pigmented and ulcerated, tumour on the palpebral side of NM	Sarcoma according to the human protocol, very lightly pigmented melanoma according to the dog-adapted protocol	Surgical resection of the mass, resection of NM at 2nd surgery	4 months after 1st surgery/ euthanized 1,5 year later when another recurrence

Description, presentation and the different diagnosis with the human and the dog-adapted immunohistochemistry protocols used in this study, then surgical treatment and prognosis after removal of the current canine membrana nictitans tumours. y: years, F: female, OD: oculus dexter, NM: nictitating membrane

### Case 2

**Fig. 2:**
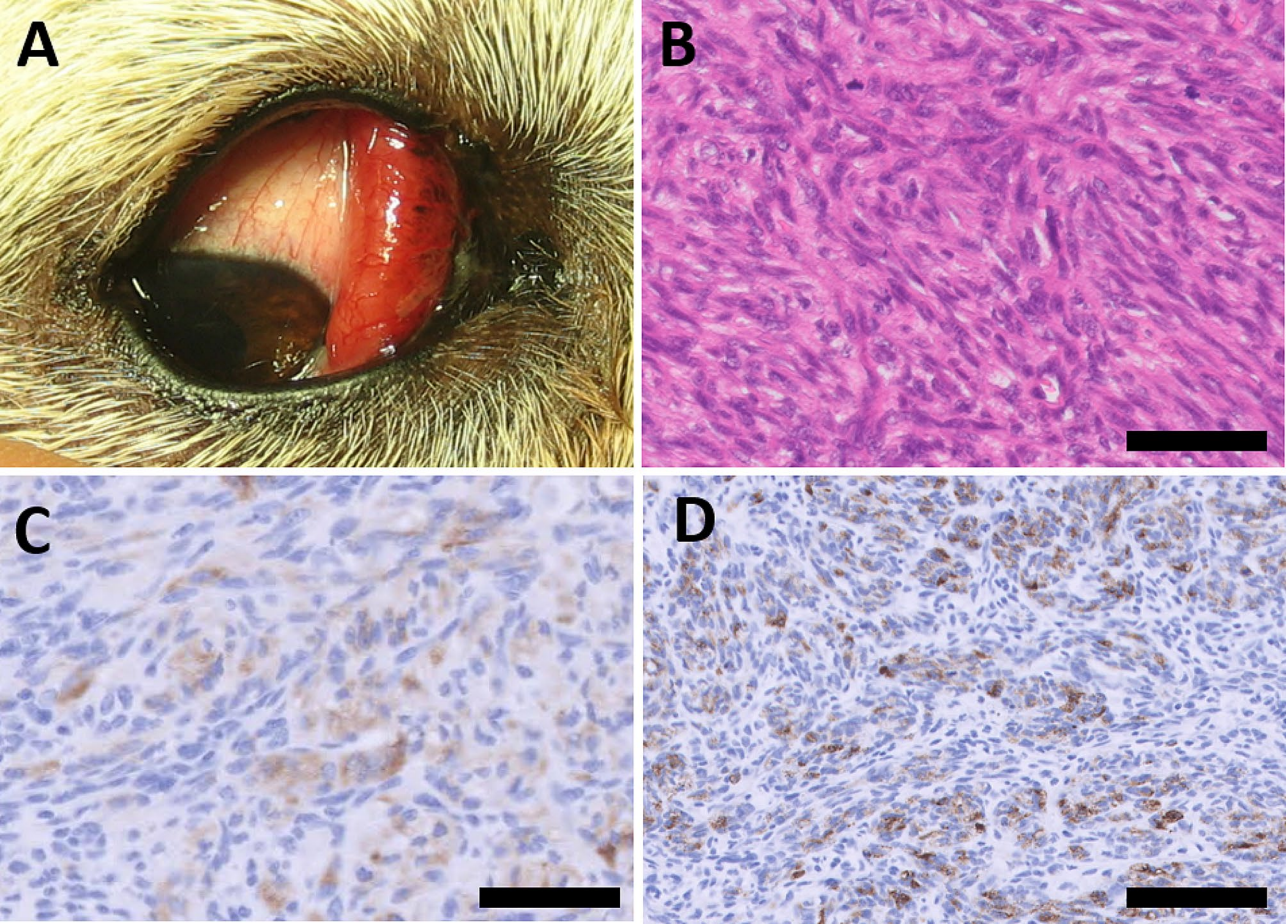
Melanoma in the nictitating membrane of a 12-year-old Akita. (**a**) Melanoma in situ before first resection. The tumour arises from the palpebral side of the nictitating membrane of the right eye and has a firm hyperemic appearance. (**b**) A population of spindle cells forming irregular interlacing fascicles is present, with numerous scattered mitotic figures present in the field. Hematoxylin and eosin (bar = 200 μm). (**c**) Immunohistochemical stain for Melan-A showing moderate cytoplasmic staining of the neoplastic cells (bar = 200 μm). (**d**) Immunohistochemical stain for PNL2 showing robust cytoplasmic staining of neoplastic cells (bar = 100 μm)

A 37.5 kg, 12-year-old intact female white coated Akita dog presented with a hyperaemic, grossly nonpigmented tumour arising from the palpebral surface of the right nictitating membrane (Fig. 2a). The ophthalmic examination was otherwise normal. The firm hyperaemic mass and part of the nictitating membrane was resected and sent for histopathological examination. A plain chest radiograph was performed which revealed multiple small nodular densities suspected to be metastases, with age-related changes as a differential diagnosis. A year and a half later, a regrowth of the tumour was observed in the nictitating membrane of the medial canthus of the right eye. An additional chest radiograph at that time showed no further development in the nodular densities. A larger resection of the nictitating membrane including the tumour was performed under general anaesthesia. The histopathological diagnosis confirmed the suspicion of recurrence of the resected tumour. Seven months later the dog had exophthalmos with exotropia and a mass in the medial canthus. A routine blood profile revealed lymphopenia and slightly elevated serum calcium. As the dog had exophthalmos and the owner did not want to euthanize the dog, an exenteration was performed. During this surgery the mass, which was now involving the medial and retrobulbar area of the orbit, was resected. Within four months there was recurrence in the medial aspect of the cutaneous scar. This area was resected, but within one month the dog had developed respiratory signs with coughing and was euthanized. Necropsy was not performed. (Table 2).

### Case 3

**Fig. 3:**
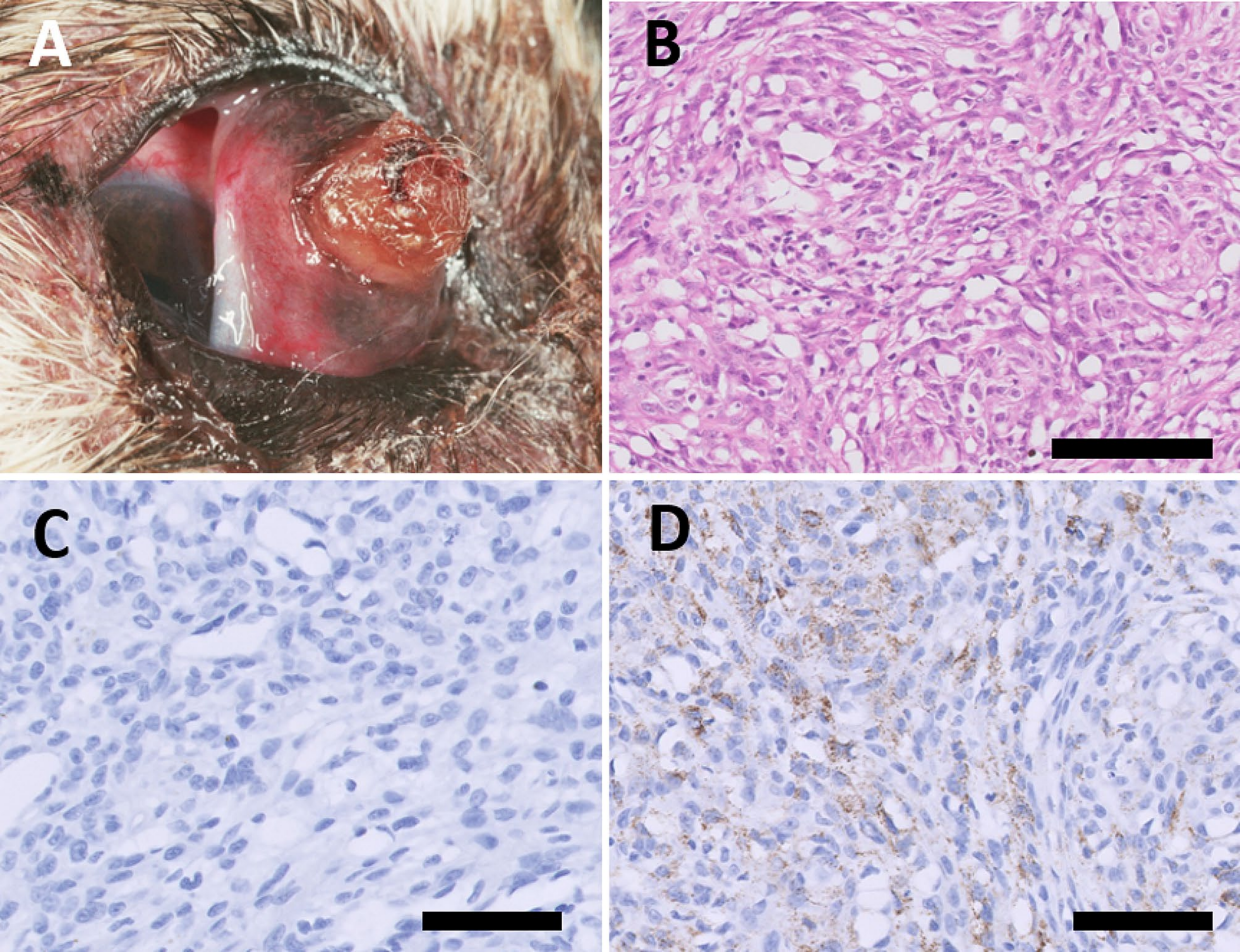
Melanoma in the nictitating membrane of a 14-year-old Shetland Sheepdog. (**a**) Melanoma in situ before second resection. The tumour arises from the palpebral surface of the membrana nictitans of the right eye and is pedunculated and ulcerated. (**b**) Neoplastic cells form short interlacing bundles and aggregates with sparse fibrovascular stroma admixed. Hematoxylin and eosin (bar = 200 μm). (**c**) Immunohistochemical stain for Melan-A is negative in this tumour (bar = 100 μm). (**d**) Immunohistochemical stain for PNL2 showing moderate cytoplasmic staining of neoplastic cells (bar = 200 μm)

A 9 kg, 14-year-old intact female tricolour Shetland Sheepdog presented with an on the surface partly pigmented pendulous tumour on the palpebral surface of the nictitating membrane of the right eye. The tumour was ulcerated which was suspected to be due to self-trauma. The ophthalmological examination was otherwise normal. There was no evidence of systemic disease on the general physical examination. Blood count and routine biochemistry were both normal. The tumour was initially resected under local anaesthesia by a veterinary ophthalmologist. Four months after excision the tumour recurred (Fig. 3a). The dog was then fully anesthetized and large areas of the nictitating membrane were surgically removed and sent for histopathological examination. The dog survived for another 1.5 years and was then euthanized because of age and regrowth of the tumour. Necropsy was not performed. (Table 2).

### Clinical findings

All three dogs were female with a mean age of 11 years at the time of the first examination. All three tumours initially appeared as a protrusion on the right side with a firm or multilobulated hyperaemic mass swelling on the palpebral surface of the nictitating membrane. In two cases the tumour was grossly nonpigmented, the third being slightly pigmented on the surface and ulcerated. All three tumours were surgically resected. All three tumours recurred after the first surgery and two of the three recurred after a second surgery. One of two recurred after a third surgery even though exenteration was performed. In the two other cases that developed recurrences, the nictitating membrane had been resected and this extended the time to the next recurrence.

### Histopathological findings

#### Case 1

(third resection) had a well-demarcated, non-encapsulated and expansile highly cellular mass that was expanding the conjunctival substantia propria adjacent to the leading edge on the palpebral surface of the right nictitating membrane (Fig. 1b), thereby mildly compressing the adjacent gland. The mass was composed of plump spindle cells that frequently formed vascular lumina where erythrocytes were present (Fig. 1c). Cells showed prominent nucleoli and there were 11 mitotic figures/standard area of 2.37 mm^2^ (corresponding to 10 HPF (high power fields). The mass was not present at the surgical margins.

#### Case 2

(first resection) presented with pleomorphic spindle tumour cells in a fascicular pattern expanding the conjunctival substantia propria (Fig. 2b), multifocally abutting on and elevating the epithelium on the palpebral surface of the right nictitating membrane. The mass was moderately demarcated, nonencapsulated, was expansile and moderately infiltrative at the periphery. Cells were pleomorphic, exhibiting karyomegaly and occasionally contained coarsely granular brown pigment in their cytoplasm. There were 8 mitotic figures/standard area of 2.37 mm^2^ (corresponding to 10 HPF). Occasional intraepithelial nests of atypical, variably pigmented cells were noted (junctional activity). The mass was present at the surgical margins.

#### Case 3

(second resection) presented with a fibrillary infiltration of pleomorphic tumour cells expanding the conjunctival substantia propria at the leading edge of the palpebral surface of the right nictitating membrane. Tumour cells were plump spindle and multifocally showed a vacuolated cytoplasm (Fig. 3b). Rare cells showed sparse coarsely granular brown pigment in their cytoplasm. There were 11 mitotic figures/standard area of 2.37 mm^2^ (corresponding to 10 HPF). The surgical margin was not affected, the narrowest margin was 3 mm.

### Immunohistochemical findings

The immunohistochemistry revealed positive staining for vimentin in all three cases, especially in case 2 where it was strongly positive. S-100 was also positive in all three cases though only sparsely-moderately in case 1 and 3, and strongly positive in 80% of the cells in case 2. SMA was slightly positive in neoplastic cells of case 3 and strongly positive in 50% of the neoplastic cells in case 2. Cytokeratin staining was negative in the tumour cells of all the three cases. GFAP was positive in both case 2 and 3, though specifically strongly in case 3. All cases were concluded to be negative when staining for Melan-A according to the human protocol. With the dog-adapted protocol Melan-A was moderately positive in approximately 30% of the cells of case 2, with appropriate cytoplasmic staining (Fig. 2c). In case 3, Melan-A showed occasional cells with strong cytoplasmic staining, representing less than 10% of the cells in section (Fig. 3c). PNL2 showed strong cytoplasmic positivity in approximately 20% of the surface of the tumour from case 2 (Fig. 2d). In the tumour from case 3 PNL2 showed moderate to strong cytoplasmic staining in approximately 50% of the surface of the neoplasm (Fig. 3d). (Table 3).

**Table 3:** Immunohistochemistry results and diagnoses

Immunohistochemistry	Case 1	Case 2	Case 3
*Vimentin*	+	++	+
*S-100*	+	80% ++	+
*SMA*	N/A	50% ++	+
*Cytokeratin*	-	-	-
*GFAP*	N/A	+	++
*Melan-A (human protocol)*	-	-	-
First diagnosis	Hemangiosarcoma	Sarcoma	Sarcoma
*Melan-A (dog-adapted protocol)*	N/A	30% +	< 10% +
*PLN2*	N/A	20% +	50% ++
Final diagnosis	Hemangiosarcoma	Lightly pigmented melanoma	Very lightly pigmented melanoma

Immunohistochemistry results and diagnoses of the canine membrana nictitans tumours by the human protocol and the dog-adapted protocol used in the current study. +: slightly positive (when estimated with a percentage this is noted), ++: strongly positive (with a percentage when noted), -: negative, N/A: not available

### Diagnosis

#### Case 1

had a classical pattern for hemangiosarcoma. Case 2 and 3 were at first diagnosed as sarcomas according to the human protocol, but after performing immunohistochemistry with dog-adapted protocols finally diagnosed as lightly pigmented melanomas. (Table 2).

### Prognoses

All three patients underwent several surgeries but had recurrence when euthanized within 22–40 months after the first surgery. One of these dogs showed systemic signs with coughing, suggesting potential metastatic disease, when euthanized 30 months after the first surgery. (Table 2).

## Discussion

In this series of malignant spindle cell tumours from the nictitating membrane we include a hemangiosarcoma, which is quite uncommon in the Nordic countries, and two lightly pigmented melanomas which are even more rarely seen [9, 10] [C. R. Reilly et al., ACVO 2005 abstract no.: 39]. We used dog-adapted immunohistochemical protocols to secure a correct diagnosis.

The breeds involved in this study were a 12 kg Dachshund, a 37,5 kg Akita, and a 9 kg Shetland Sheepdog. The Dachshund with the hemangiosarcoma corresponded well to what is recorded in former studies on nictitating membrane hemangiosarcoma where there is no apparent breed disposition, but middle and large-size dogs are more commonly affected [9, 10, 34]. The white coated Akita and the tricolour Shetland Sheepdog with the melanomas were not black coated as former studies have indicated, but on the other hand these tumours were only very sparsely pigmented. All three patients in this study were intact females, which may be an effect of the low numbers of cases included in this report, as most former studies on conjunctival hemangiosarcoma and melanomas demonstrated no sex predilection or a slight male predominance [3, 9, 10] [J. W. Herrmann et al., ACVO 2016 abstract no.: 123]. Only one study suggested a female predominance in conjunctival melanomas [8]. This is even contrary to the studies that have suggested a hormonal link between hemangiosarcoma in general and neutering status, where occurrence of hemangiosarcomas is more common in neutered individuals [34]. The age at onset was 8–14 years (mean 11 years), which correlates well with the age span reported in former studies [3, 8–10].

The clinical signs of protrusion of the nictitating membrane with firm or irregular masses of different shades of pink, expanding the nictitating membrane most often in the nonpigmented areas, epiphora and/or conjunctival hyperaemia are as described in former studies on nictitating membrane neoplasia [2, 6, 9–11, 14–24]. The blood blister-like appearance is quite typical for hemangiosarcomas [34]. The ulcerated surface of the melanoma in case 3 could be an indication that the tumour was becoming devitalized or a sign of self-trauma. All three cases were positioned on the palpebral surface of the nictitating membrane. In former studies tumour growth in general has been reported on the bulbar surface, the palpebral surface or from the leading edge of the nictitating membrane [4]. Hemangiosarcomas and hemangiomas seem to originate more often from either the palpebral surface or the leading edge of the nictitating membrane [9, 10, 33]. In this case series the hemangiosarcoma arose on the palpebral surface of a sparsely pigmented nictitating membrane, correlating with the theory that there is an increased risk factor for developing vascular neoplasia when the nonpigmented nictitating membrane is exposed to UV light [10, 31–33]. No evidence of actinic damage (solar elastosis, solar vasculopathy or solar fibrosis) was noted around the neoplasms; however, these lesions are not always visible around UV-induced tumours. As a prevention one could therefore make sure to have limited sun exposure for their dog, even though the UV index in Scandinavia according to WHO is only around half of what it is in Southern Europe, in Australia and in USA [48].

Both the hemangiosarcoma and the melanomas recurred one or several times after the first surgery. Sarcomas in general and melanomas both demonstrate aggressive, locally invasive tissue involvement. Some studies on hemangiosarcomas even discuss de novo tumours arising from the same location [33]. Early, complete surgical excision of hemangiosarcomas is recommended and may be curative, though recurrence is a risk [10, 33]. Melanomas have the potential to occur and recur multifocally with recurrence being a risk even after aggressive surgical treatment like a complete excision of the nictitating membrane [C. R. Reilly et al., ACVO 2005 abstract no.: 39]. Full-thickness resection of the whole nictitating membrane seemed to delay recurrence by at least 1.5 years in this case series.

The treatment of choice for hemangiosarcomas and melanomas is surgery. The prognosis is probably better when surgery is performed by a surgeon with microsurgical skills and access to an operating microscope and microsurgical equipment [33]. Radiation therapy and systemic chemotherapy has been used with success in non-dermal hemangiosarcomas [34]. For the melanomas, both radiation therapy, systemic chemotherapy and immunotherapy is proven to be successful [43–45]. None of the above-mentioned therapies were applied to the three cases in this series.

All the three cases showed recurrence when euthanized 22–40 months after the first surgery. The presence of respiratory signs in case 2 indicated possible metastatic spread, which is also described in earlier studies, although metastasis was not confirmed in this case [8].

The three cases were diagnosed by histopathology and immunohistochemistry. Immunohistochemistry has in the recent years been used in several studies on tumours of the nictitating membrane [14, 16, 18–22, 24]. It is a valuable tool for the pathologist to further differentiate the type of tumour being analysed and to diagnose more uncommon tumours and new subtypes giving us new knowledge. It is essential to use dog-adapted protocols to get a precise diagnosis [46, 49]. These dog-adapted immunohistochemical protocols secure a correct antigen retrieval and thereby a correct diagnosis of the tumour. Antigen retrieval is a technique required in most formalin-fixed tissues before immunohistochemical staining. It is used to reverse epitope masking and restore epitope-antibody binding often lost during the fixation process. Avoiding this step may result in weak or false negative staining [49].

## Conclusions

We report three rare cases of spindle cell tumours in the nictitating membrane in dogs. The tumours arose in nonpigmented areas of membrana nictitans and they showed invasive growth with post-surgical recurrence in all three dogs, once or several times. We performed extensive histopathological and immunohistochemical investigations to further subclassify these tumours. It is essential to use dog-adapted immunohistochemical protocols to reach the correct diagnosis.

## Data Availability

The materials analysed during the current study are available from the corresponding author on reasonable request.

## Declarations

### Prior publication

Data has not been published previously.

### Authors’ information

This publication is part of the specialist training program for KBK to become a veterinary specialist in ophthalmology.

## References

[1] Krehbiel JD, Langham RF (1975). Eyelid neoplasms of dogs. Am J Vet Res.

[2] Wilcock B, Peiffer RJ (1988). Adenocarcinoma of the gland of the third eyelid in seven dogs. J Am Vet Med Assoc.

[3] Schäffer EH, Pfleghaar S, Gordon S, Knodlseder M (1994). Malignant nictitating membrane tumors in dogs and cats. Tierarztl Prax.

[4] Labelle AL, Labelle P (2013). Canine ocular neoplasia: a review. Vet Ophthalmol.

[5] Dees DD, Schobert CS, Dubielzig RR, Stein TJ (2016). Third eyelid gland neoplasms of dogs and cats: a retrospective histopathologic study of 145 cases. Vet Ophthalmol.

[6] Lavach JD, Snyder SP (1984). Squamous cell carcinoma of the third eyelid in a dog. J Am Vet Med Assoc.

[7] Collier LL, Collins BK (1994). Excision and cryosurgical ablation of severe periocular papillomatosis in a dog. J Am Vet Med Assoc.

[8] Collins BK, Collier LL, Miller MA, Linton LL (1993). Biologic behavior and histologic characteristics of canine conjunctival melanoma. Prog Vet Comp Ophthalmol.

[9] Liapis IK, Genovese L (2004). Hemangiosarcoma of the third eyelid in a dog. Vet Ophthalmol.

[10] Pirie CG, Knollinger AM, Thomas CB, Dubielzig RR (2006). Canine conjunctival hemangioma and hemangiosarcoma: a retrospective evaluation of 108 cases (1989–2004). Vet Ophthalmol.

[11] Peiffer RLJ, Duncan J, Terrell T (1978). Hemangioma of the nictitating membrane in a dog. J Am Vet Med Assoc.

[12] Buyukmihci N, Stannard AA (1981). Canine conjunctival angiokeratomas. J Am Vet Med Assoc.

[13] George C, Summers BA (1990). Angiokeratoma: a benign vascular tumour of the dog. JSAP.

[14] Mathes RL, Noble SJ, Ellis AE (2016). Leiomyoma of the third eyelid in a dog. Vet Ophthalmol.

[15] Hallstrom M (1970). Mastocytoma in the third eyelid of a dog. J Small Anim Pract.

[16] Hong IH, Bae SH, Lee SG, Park JK, Ji AR, Ki MR (2011). Mucosa-associated lymphoid tissue lymphoma of the third eyelid conjunctiva in a dog. Vet Ophthalmol.

[17] Holm F, Hardon T, Clasen-Linde E, Hjorth Mikkelsen L, Heegaard S (2018). B-cell lymphoblastic lymphoma of the nictitating membrane as the first presenting sign in a 2-year-old Springer Spaniel. Clin Case Rep.

[18] Perlmann E, Dagli MLZ, Martins MC, Siqueira SAC, Barros PSM (2009). Extramedullary plasmacytoma of the third eyelid gland in a dog. Vet Ophthalmol.

[19] Bondoc A, Izawa T, Hirata S, Hasegawa T, Kuwamura M, Golbar HM (2014). Myoepithelioma of the gland of the third eyelid in a dog. J Comp Pathol.

[20] Miyazaki A, Yonemaru K, Hirata A, Yanai T, Sakai H (2015). Histopathological and immunohistochemical features of atypical epithelial tumours of the gland of the third eyelid in seven dogs. J Comp Pathol.

[21] Rodriguez Galarza RM, Shrader SM, Koehler JW, Abarca E (2016). A case of basal cell carcinoma of the nictitating membrane in a dog. Clin Case Rep.

[22] Sano Y, Miyazaki M, Yaegashi R, Okamoto M, Masuko A, Maehara S (2019). Basal cell adenocarcinoma on bulbar conjunctiva of third eyelid in a dog. J Vet Med Sci.

[23] Milo J, Snead E (2014). A case of ocular canine transmissible venereal tumor. Can Vet J.

[24] vom Hagen F, Romkes G, Kershaw O, Eule JC (2015). Malignant peripheral nerve sheath tumor of the third eyelid in a 3-year-old rhodesian ridgeback. Clin Case Rep.

[25] Goldschmidt MH, Goldschmidt KH, Meuten DJ (2017). Chapter 4 - epithelial and melanocytic tumors of the skin. Tumors in domestic animals. Fifth edit.

[26] Etchevers HC, Dupin E, Le Douarin NM (2019). The diverse neural crest: from embryology to human pathology. Development.

[27] Mark Elwood J, Jopson J (1997). Melanoma and sun exposure: an overview of published studies. Int J Cancer.

[28] Vicente ALSA, Novoloaca A, Cahais V, Awada Z, Cuenin C, Spitz N (2022). Cutaneous and acral melanoma cross-OMICs reveals prognostic cancer drivers associated with pathobiology and ultraviolet exposure. Nat Commun.

[29] Gillard M, Cadieu E, De Brito C, Abadie J, Vergier B, Devauchelle P (2014). Naturally occurring melanomas in dogs as models for non-UV pathways of human melanomas. Pigment Cell Melanoma Res.

[30] Mullin C, Clifford CA, Vail DM, Thamm DH, Liptak JM (2020). Chapter 34 - miscellaneous tumors. Withrow & MacEwen’s Small Animal Clinical Oncology. Sixth edit.

[31] Hargis AM, Lee AC, Thomassen RW (1978). Tumor and tumor-like lesions of perilimbal conjunctiva in laboratory dogs. J Am Vet Med Assoc.

[32] Nikula KJ, Benjamin SA, Angleton GM, Saunders WJ, Lee AC (1992). Ultraviolet radiation, solar dermatosis, and cutaneous neoplasia in beagle dogs. Radiat Res.

[33] Richardson S, Deykin AR (2021). Surgical treatment of conjunctival hemangioma and hemangiosarcoma: a retrospective study of 52 dogs. Vet Ophthalmol.

[34] Mullin C, Clifford CA (2019). Histiocytic sarcoma and hemangiosarcoma update. Vet Clin North Am Small Anim Pract.

[35] Dubielzig RR, Ketring K, McLellan GJ, Albert DM (2010). Diseases of the eyelids and conjunctiva. Veterinary Ocular Pathology: a comparative review.

[36] Bray JP (2016). Soft tissue sarcoma in the dog– part 1: a current review. JSAP.

[37] Casali PG, Abecassis N, Aro HT, Bauer S, Biagini R, Bielack S (2018). Soft tissue and visceral sarcomas: ESMO-EURACAN clinical practice guidelines for diagnosis, treatment and follow-up. Ann Oncol.

[38] von Mehren M, Randall RL, Benjamin RS, Boles S, Bui MM, Ganjoo KN (2018). Soft tissue sarcoma, version 2.2018, NCCN clinical practice guidelines in oncology. JNCCN.

[39] Ethun CG, Delman KA (2016). The importance of surgical margins in melanoma. J Surg Oncol.

[40] Coit DG, Thompson JA, Algazi A, Andtbacka R, Bichakjian CK, Carson WE (2016). Melanoma, version 2.2016, NCCN clinical practice guidelines in oncology. JNCCN.

[41] Selmic LE, Samuelson J, Reagan JK, Mesa KJ, Driskell E, Li J (2019). Intra-operative imaging of surgical margins of canine soft tissue sarcoma using optical coherence tomography. Vet Comp Oncol.

[42] MacArthur KM, Baumann BC, Sobanko JF, Etzkorn JR, Shin TM, Higgins HW (2021). Compliance with sentinel lymph node biopsy guidelines for invasive melanomas treated with Mohs micrographic surgery. Cancer.

[43] Bergman PJ, Selmic LE, Kent MS, Vail DM, Thamm DH, Liptak JM (2020). Chapter 20 - melanoma. Withrow & MacEwen’s Small Animal Clinical Oncology. Sixth edit.

[44] Ralli M, Botticelli A, Visconti IC, Angeletti D, Fiore M, Marchetti P (2020). Immunotherapy in the treatment of metastatic melanoma: current knowledge and future directions. J Immunol Res.

[45] Westberg S, Sadeghi A, Svensson E, Segall T, Dimopoulou M, Korsgren O (2013). Treatment efficacy and immune stimulation by AdCD40L gene therapy of spontaneous canine malignant melanoma. J Immunother.

[46] Ramos-Vara JA, Miller MA (2011). Immunohistochemical identification of canine melanocytic neoplasms with antibodies to melanocytic antigen PNL2 and tyrosinase: comparison with melan A. Vet Pathol.

[47] Mikkelsen LH, Andreasen S, Melchior LC, Persson M, Andersen JD, Pereira V (2017). Genomic and immunohistochemical characterisation of a lacrimal gland oncocytoma and review of literature. Oncol Lett.

[48] Global Health Observatory data repository. Exposure to solar ultraviolet (UV) radiation - data by country. https://apps.who.int/gho/data/view.main.35300. 2004. Accessed 25 August 2023.

[49] Ramos-Vara JA (2005). Technical aspects of immunohistochemistry. Vet Pathol.

